# Diagnosis of ACL and meniscal injuries: MR imaging of knee flexion versus extension compared to arthroscopy

**DOI:** 10.1186/2193-1801-2-213

**Published:** 2013-05-08

**Authors:** Claus Muhle, Joong Mo Ahn, Constanze Dieke

**Affiliations:** Radiology Vechta, St. Marienhospital Vechta, Marienstr. 6-8, 49377 Vechta, Germany; University of Pittsburgh, 3950 Presby South Tower 200, Lothrop Street, Pittsburgh, PA 15213 USA

**Keywords:** MRI, Knee, Anterior cruciate ligament, Meniscus

## Abstract

The aim of the study was to evaluate whether MR Imaging of the knee at 30° and 55° of flexion can improve the diagnosis of anterior cruciate ligament and menisci injuries compared to arthroscopy and imaging during extension of the knee joint. Knee joints from 40 patients with clinical suspicion of an anterior cruciate ligament (ACL) rupture were examined using MRI while the knee joint was either extended or flexed at 30° and 55° of knee flexion. A standard MR knee coil was used at extension, whereas at 30° and 55° of flexion a non-metallic positioning device and a flexible surface coil was placed ventral to the patella. Sagittal T2-weighted TSE sequences were acquired. In 29 of 40 patients, arthroscopy results were compared to the MRI examinations. Image quality of MRI examinations was evaluated using a three-point rating scale in a blinded fashion. Images were compared between groups and rated as better quality, same quality, or worse quality. Additionally, each angle MRI was compared to arthroscopy results. Partial ACL ruptures were diagnosed with 63% accuracy using MR imaging at 30° and 55° of knee flexion compared to 50% accuracy during knee extension. MRI imaging of complete ACL ruptures resulted in 83% accuracy of diagnosis when imaged at 30° flexion, 93% accuracy at 55° flexion, and 83% accuracy at extension. The accuracy of diagnosing medial meniscus lesions was 73% at extension, 64% at 30° flexion and 73% at 55° of flexion. MR imaging was only able to diagnose lateral meniscus tears with 55% accuracy in all three knee positions. The diagnosis of meniscal tears was more difficult due to small peripheral tears. The improved results in the diagnosis of ACL tears in response to 30° flexion and in particular in response to 55° flexion were based on the fact that the anterior cruciate ligament moved further away from the intercondylar roof with increased knee flexion. During flexion the ligament tension decreased, which causes the anterior cruciate ligament to have cylindrical shape and therefore made visualization of the injury easier. In conclusion, MR Imaging of the knee at 55° of flexion and less at 30° of flexion allows an improved diagnosis of injuries to the anterior cruciate ligament as compared to MRI examinations at extension. The diagnosis of meniscal injuries, however, was not superior at both flexion positions compared to commonly performed examinations at knee extension.

## Introduction

Anterior cruciate ligament (ACL) tear is currently the most common ligament injury to the knee joint, occurring in as many as 1 in 3,500 individuals each year (Barber-Westin & Noyes 2011). An ACL tear typically includes valgus-flexion, and external rotation. This type of injury results in rupture of the medial collateral ligament, the dorsomedial capsule including the posterior horn of the medial meniscus, and the anterior cruciate ligament (Barry et al. 1996; De Smet & Graf 1994; Duncan et al. 1995; Stäbler & Freyschmidt 2005; Korn et al. 2011; Subhas et al. 2011).

The loss of function of the ACL causes into an anteromedial joint instability, where the tibial head has increased mobility relative to the thigh. Changes in the kinematics of the knee joint leads to an increased stress on the menisci, whereby increased damages to the cartilage can be observed over several years (Logan et al. 2004; Arnoldi et al. 2011). Surveys have also found that meniscal injures accompany injuries to the ACL in up to one third of cases (Katz & Weitzel 2009).

MRI scanning of the knee joint is routinely prescribed as the initial non-invasive diagnosis tool after clinical examination, which can include the “anterior drawer test” and “Lachman test”.

MR Imaging is the method of choice to further evaluate additional knee injuries that may accompany an ACL tear (Fritz 2003; Muhle et al. 1996; Niitsu et al. 1990; Niitsu et al. 1996; Pereira et al. 1998; Kam et al. 2010). Despite a technically flawless MR exam, the images can be difficult to interpret and are observer dependent. Because of these issues, false negative diagnostic findings are to be expected in up to 20% of patients regardless of the experience of the examiner. This is mainly the case in the diagnosis of injuries to the cruciate ligament and to the meniscus and possible sources of error may vary (Barry et al. 1996; De Smet & Graf 1994; De Smet & Graf Fritz 2003; Brandser et al. 1996; De Smet et al. 1994; Falchook et al. 1996; Justice & Quinn 1995; Kreitner et al. 1998; Liu et al. 1995; McCauley et al. 1994; Roychowdhury et al. 1997; Umans et al. 1995; Vahey et al. 1990; Van Dyck et al. 2012; Van Dyck et al. 2011).

It has been reported that MR imaging the knee during flexion may permit better visualization of the ACL. These studies examined MR imaging while the knee was positioned at a 45° and compared to imaging during full extension of the knee joint. Results indicated better diagnosis of the ACL rupture at the flexed position (Niitsu et al. 1996; Pereira et al. 1998).

In further studies, it was not possible to make clear statements about an improved meniscus diagnosis in response to flexion compared to extension (Niitsu et al. 1991; Niitsu et al. 1988; Niitsu et al. 2000). However, a comparison of MR imaging studies with arthroscopic results has not yet been carried out until now.

Therefore, the purpose of this study was to evaluate whether MR scans at a flexed knee position provide more accurate diagnosis of ACL and meniscus injuries than MR examinations obtained at full extension compared to knee arthroscopy (Niitsu et al. 1996; Pereira et al. 1998).

## Material and methods

### Patients

Recruitment consisted of patients with acute knee traumas. 40 patients (11 women and 29 men; 16 left and 24 right knees) between the ages of 16 and 63 (average age 35 years) with a clinical suspicion of an ACL rupture were included in this study. Between August 2009 and November 2011 surgeons and orthopedists referred patients that were 1–9 days post injury. The patients were sent to us by surgeons and orthopedists during the period between August 2009 and February 2012. Written informed consent was obtained after explanation of the IRB approved study.

### MR imaging

The examinations were carried out in a Siemens 1.5 Tesla Magnetom-Symphony whole-body MRI scanner (Erlangen, Germany). Sagittal scans of the extended knee (0°) joint were obtained while patients were in supine position using a standard.

Siemens knee coil. The leg was rotated slightly outwardly. It is known that an external rotation of the leg by 10-15° makes it possible to visualize the entire course of the anterior cruciate ligament on sagittal images (Stäbler & Freyschmidt 2005; Reiser et al. 1992; Reiser & Semmler 2002).

### Positioning device

**Figure 1:**
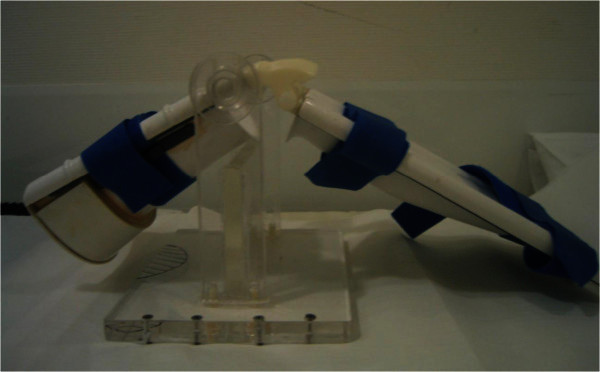
**Positioning device made from nonferromagnetic materials, which was attached to the patient’s table.** The positioning device consists of two supports for the thigh and lower leg, which are connected to each other by a common axis of rotation. In supine position, the lower leg and thigh are attached to the supports with Velcro straps to avoid displacement of the knee during knee extension and flexion.

In order to obtain images during knee flexion and extension a special positioning device was created using nonferromagnetic materials (Figure 1) and attached to the scanner table. It consisted of two supports for the thigh and lower leg, which were connected to each other by a common axis of rotation. The device could be used for either leg by means of a simple modification. In the supine position, the lower leg and thigh were attached to the supports with Velcro straps to avoid displacement during knee extension and flexion. It was possible to manually adjust the movable bar portion, in which the lower leg was positioned, via a hinge joint, so as to bring the knee joint into the desired flexed positions of 30° and 55°. The knees were positioned so that the extension-flexion axis was identical to the revolving axis of the positioning device. For signal reception at knee flexion, we positioned two ring-shaped flexible surface coils 15 cm in each diameter on both sides of the knee, so that it encircled the whole knee. The scan parameters are listed in Table 1.

**Table 1:** **MR imaging protocols**

Knie position	Sequence	Orientation	Slices	TR (msec)	TE (msec)	T/G (mm)	FOV (cm)	Matrix	Scan time
Extension (0°)	TSE FS-PD	axial	28	2900	20	3/1	19	256x224	3:52
	TSE FS-PD	coronal	28	2900	20	3/1	19	256x224	3:52
	T1-SE	sagittal	28	650	20	3/1	19	256x224	2:37
	TSE FS-PD	sagittal	28	2900	20	3/1	19	256x224	3:52
30° Flexion	TSE FS-PD	sagittal	28	2900	20	3/1	19	256x224	3:52
55° Flexion	TSE FS-PD	sagittal	28	2900	20	3/1	19	256x224	3:52

Note.- FA = flip angle, G = intersection gap, NA = not applicable, NSA = number of signals acquired, PD = proton density-weighted, T = section thickness, TE = echo time, TR = repetition time, T 1 = T1-weighted, T2 = T2-weighted.

### Arthroscopy

In 29 patients an arthroscopy of the injured knee joint was carried out. The interval between MRI examination and arthroscopy was 1–6 weeks (on average: 4 weeks). In the case of arthroscopy, an anterolateral access was chosen, so that it was possible to evaluate all of the compartments (medial, lateral, retro-patellar) of the knee joint by means of a palpating hook. The findings were noted in the surgical report.

### Evaluation

Two radiologists, having 22 years and 18 years of experience in musculoskeletal radiology blinded to the anamnetic, clinical or arthroscopic findings while evaluating the MRI images. All images were obtained and read prior in consensus to the arthroscopy procedure.

### Anterior cruciate ligament analysis

MR image quality was evaluated between groups (0° vs 30°, 0° vs 55°, 30° vs 55°) using a three-point rating scale (+ = better; 0 = the same; - = worse). The scans were compared with reference to the following portions of the anterior cruciate ligament:

1. femoral ACL end including the femoral appendage
2. Pars intermedia
3. tibial ACL end including the tibial appendage.

In addition, the torn ligament portions were evaluated (Niitsu et al. 1996; Pereira et al. 1998; Niitsu et al. 1988). Subsequently, a diagnosis of the anterior cruciate ligament was made in each knee position in response to extension (0°), 30° and 55°. It was to be evaluated thereby whether the ACL seemed to be “normal (intact)”, “partially torn” or “completely ruptured”. The MRI diagnosis was thereby based on direct, primary signs of changes to the ligament itself, and on indirect, secondary signs as detailed below.

The following signs were listed as direct signs of an ACL rupture (Stäbler & Freyschmidt 2005; Fritz 2003; Brandser et al. 1996; Falchook et al. 1996; Boeree & Ackroyd 1992; Robertson et al. 1994; Tung et al. 1993):

1. Ligament interruption, depiction of ligament fragments or a lack of depiction of the ligament.
2. Wavy course of the ligament or fibers.
3. Fluid-equivalent signal within the ligament.
4. ACL, which is diffusely signal-elevated, swollen, which can be distinguished in an unfocussed manner.
5. Abnormally inclined course of ligament portions with a clear deviation from the Blumensaat’s line.

Listed as indirect signs of an ACL rupture were (Stäbler & Freyschmidt 2005; Katz & Weitzel 2009; Kam et al. 2010; De Smet et al. 1994; Reiser & Semmler 2002; Boeree & Ackroyd 1992; Robertson et al. 1994):

1. Anterior subluxational of the tibia compared to the femur.
2. Posterior subluxational of the posterior horn of the lateral meniscus in relation to the tibia.
3. Posterior cruciate ligament extending in a partially concave manner.
4. Contusion oedema in the postero-lateral portion of the tibia and in the central to anterior portion of the lateral femoral condyle.
5. Concomitant injuries: depression fracture on the lateral femoral condyle, inner ligament lesion, meniscus tear, Segond fracture, contusions to the Hoffa fat pad.

### Medial and lateral meniscus

The same three-point rating scale (better – same – worse) comparing groups was also used to evaluate the menisci.

Diagnosis of both the medial and lateral meniscus was described as “normal”, “degeneration” and “tear”.

### Arthroscopy

In 29 of 40 patients, arthroscopy served as the “gold standard” for comparison to the MRI analysis. The findings noted in the surgical report were compared to those of the MRI analysis. Each deviation of the MRI findings from the result of the surgery was analyzed as being a false-positive or false-negative finding.

### Statistical analysis

In addition to the calculation of the sensitivity and specificity, the binomial test was carried out. A t-test, was used to calculate the confidence intervals of findings between the knee positions. Significant results were assumed on the 5% level (p = 0.05).

## Results

**Figure 2:**
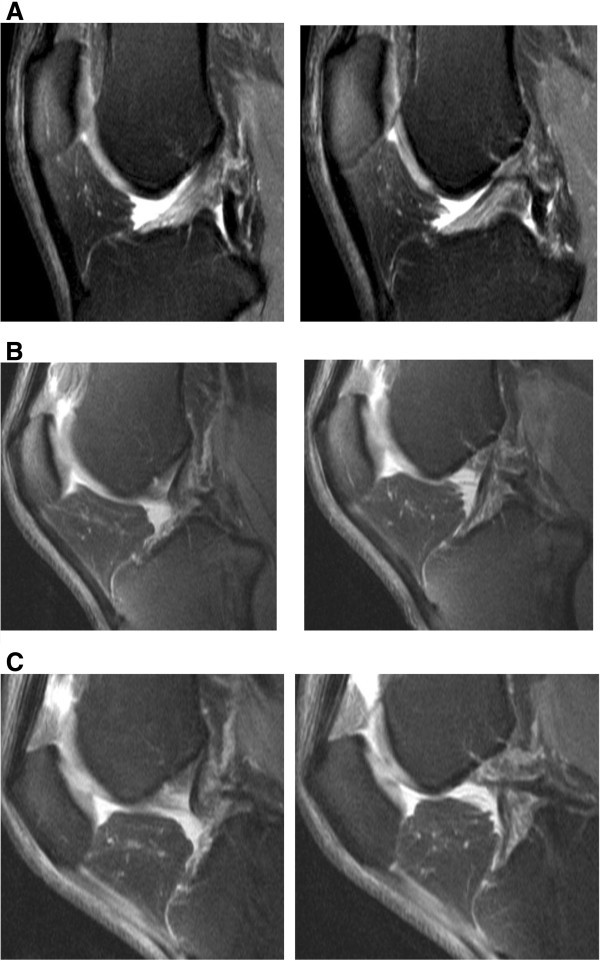
**Normal ACL images obtained at knee extension (A), 30° of knee flexion (B) and 55° of knee flexion (C) on T2-weighted TSE FS sagittal image (2900/90).** At extension (**A**) the ACL fibers are seen as linear, striate fibers extending from the roff of the femoral notch to the tibial surface. At 30° and 55° of flexion (**B**, **C**) a lengthening with astraing-like appearance of the ACL is seen. The posterior ACL, changes its appearance from an angular-konvex contour at extension (**A**), to a more straight like appearance with increased flexion angles (**B**, **C**).

In all patients, MR images at all three different knee positions (0°, 30°, 55°) showed a sufficient image quality and thus readability. The flexion angles varied between 28° and 43° in the first position and between 52° or 72.5° in the second angle position. The average was thus 30° or 55°, respectively. The different flexion angles were mainly based on the different leg lengths; in part, however, distinct articular effusions or other after-effects of the accident also existed, which, due to pain, did not allow for the setting at the same angle positions (Figure 2).

### Extension vs. 30° flexion

When comparing extension to 30° flexion, it was possible to better evaluate the femoral ACL end in 14 of 40 MRIs (35%), (Table 2). The tibial end of the ACL was better seen at 30° of flexion in 7 of 40 MR scans (18%) and the middle portion of the ACL in 11 MRI´s (28%).

**Table 2:** **MRI visualization of the ACL portions, the ACL tear and the meniscus in comparison between various knee positions**

	30° flexion vs. extension (0°)			55° flexion vs. extension (0°)			55° flexion vs. 30° flexion		
ACL	Worse n / %	Better n / %	Same n / %	Worse n / %	Better n / %	Same n / %	Worse n / %	Better n / %	Same n / %
- femoral portion	2 / 5	14 / 35	24 / 60	1 / 2	20 / 50	19 / 48	0 / 0	11 / 27	29 / 73
- midportion	4 / 10	11 / 28	25 / 62	1 / 2	22 / 55	17 / 43	1/ 2	9 / 23	30 / 75
- tibial portion	3 / 8	7 / 18	30 / 74	2 / 5	15/ 38	23 / 57	1 / 2	8 /20	31 / 78
**Torn ACL portion**	3/8	14/35	23/57	2/5	23/57	15/38	1/3	10/25	29/72
**Meniscus**									
- medial	12 / 30	4 / 10	24 / 60	8 / 20	3 / 7	29 / 73	1 / 2	8 / 20	31 / 78
- lateral	12 / 30	3 / 8	25/ 62	6/ 15	3 / 7	31 / 78	0 / 0	8 / 20	32 / 80

Abbreviations:

ACL = anterior cruciate ligament.

n = number.

% = percentage.

When compared to extension, the torn ligament ACL portion was better evaluated at 30° of flexion in 14 of 40 patients (35%). Statistically significant differences (p ≤ 0.05) in delineation of the ACL and detection of the ACL tear was observed, when comparing the MRI scans at 0° extension and at 30° flexion.

An equivalent evaluation of the medial and lateral meniscus was performed at 0° and 30°. In only 4 of 40 MRI´s (10%), the medial meniscus was better evaluated at 30° of flexion compared to extension (0°); for the lateral meniscus, evaluation at 30° was better than at extension (0°) in 3 of 40 MRI’s (8%). There were no statistically significant differences (p > 0.05), (Table 2).

### Extension vs. 55° of flexion

Visualization was rated better in 20 of 40 MR examinations (50%) of the femoral ACL end as well as 22 of 40 MR examinations (55%) of the middle ACL portion when images from extension were compared to 55° of flexion. Diagnosis of the tibial end of the ACL was better at 55° of flexion in 15 of 40 MRIs (38%). The torn ACL portions were more easily diagnosed at 55° of flexion in 23 of 40 MRI’s (57%) when compared to extension. The statistical analyzes resulted in significant differences for both, the delineation of the ACL and the detection of the torn ACL part (p ≤ 0.05). There were no significant differences (p > 0.05) in ease of diagnosis when images of meniscus tears at 55° were compared to extension.

### 30° of flexion vs. 55° of flexion

When MR images at 30° of flexion were compared to 55° of flexion, the visualization of the femoral end, the mid portion and the tibial end of the ACL was evaluated better at 55° of flexion inbetween 20 to 27% of MRI’s. A superior diagnosis of the the ACL tear was only seen only in 25% of the MRI scans (p = 0.05).

In the diagnosis of the menisci, the results for the different degrees of flexion were similar. It was not possible to observe significant differences in the menisci at both different knee positions (Table 2).

### Arthroscopy vs. MRI

An arthroscopy was performed in 29 patients an average of 4 weeks after the knee injury (range 1–6 weeks). The MRI examinations were compared to the arthroscopic results. In 11 patients, no arthroscopy was performed. In three of these 11 non -arthroscopic verified cases, an old anterior cruciate ligament rupture was diagnosed on MRI. Due to the fact that there was no clinical sign of knee instability, no arthroscopy was performed in these three patients. In two additional patients, an arthroscopy was not performed due to the older age of the patients. At three or six months follow-up, a satisfactory knee stability was seen in these two patients. The remaining six patients declined arthroscopy. After half a year of conservative therapy, three of these patients had slight knee instability, but this did not lead to any limitations at work.

### Arthroscopy diagnoses

**Figure 3:**
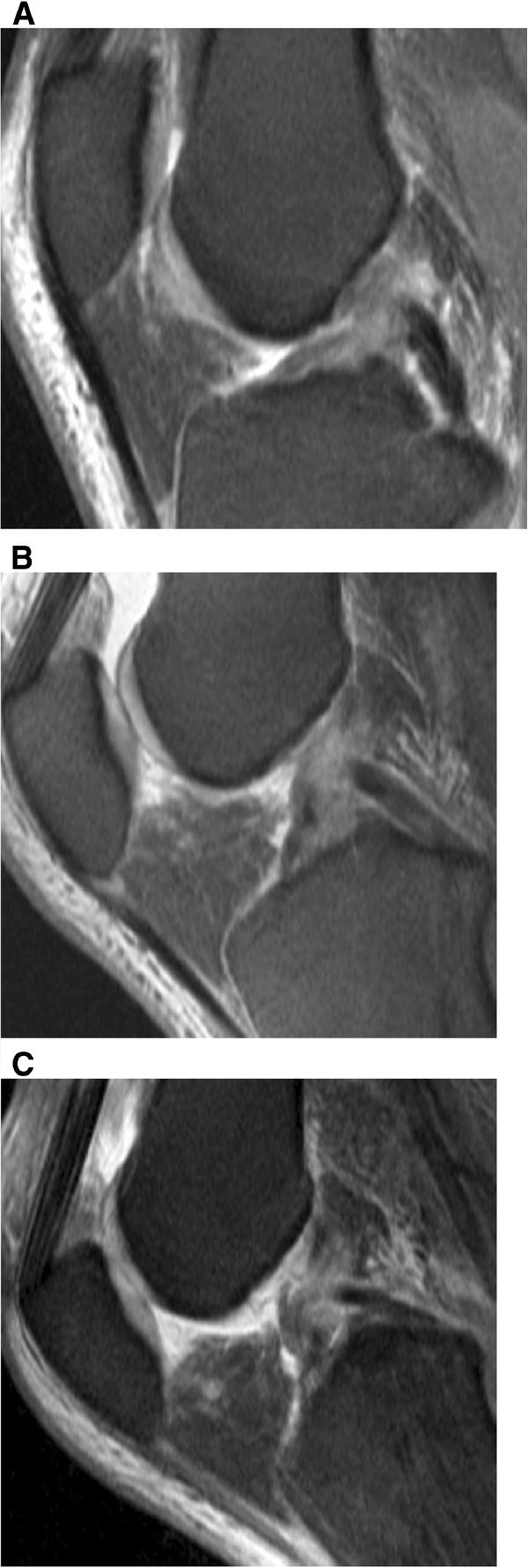
**Arthroscopically proven partial ACL-rupture in a 53-year old patient after ski accident.** (**A**) T2-weighted TSE FS sagittal image (2900/90) obtained at knee extension shows an irregularity of the midportion of the ACL. On T2-weighted TSE FS sagittal images (2900/90) at 30° of knee flexion (**B**) and at 55° of knee flexion (**C**) the partial continuity of the ACL bundles are better recognized than on MR images taken at knee extension (**A**).

**Figure 4:**
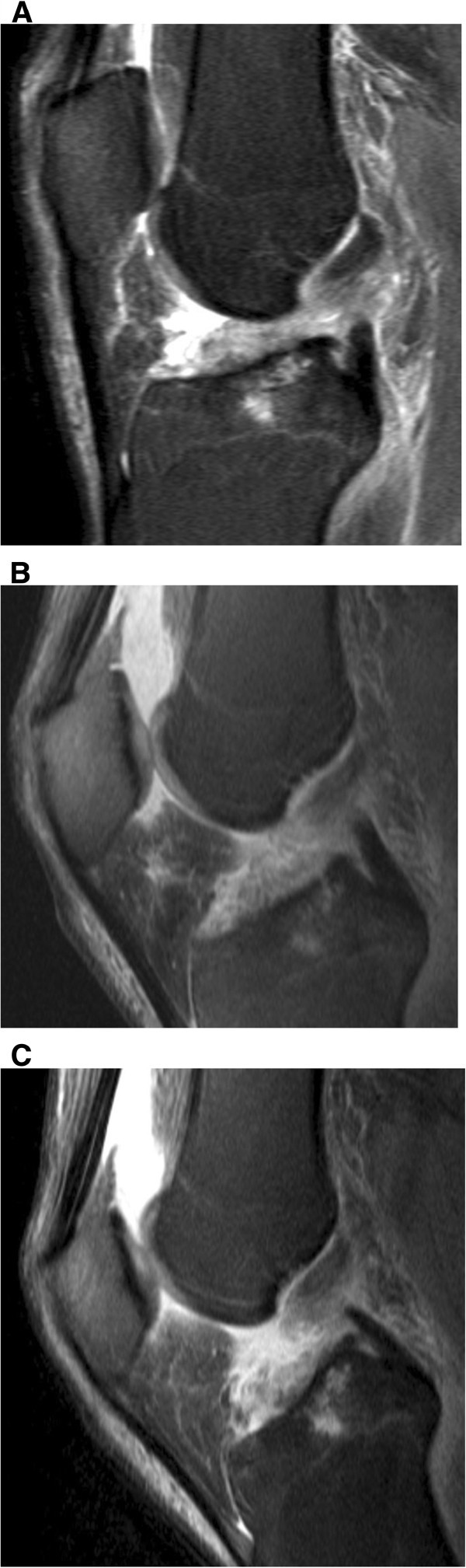
**Complete ACL rupture in a 50-year old patient.** T2-weighted TSE FS sagittal image (2900/90) obtained at extension (**A**), 30° (**B**), and 55° (**C**) of knee flexion better demonstrates the complete disruption and retraction of the torn ACL fibers than MR images taken at knee extension (**A**). At knee extension an accurate differentiation between a partial and complete ACL rupture is not possible.

**Figure 5:**
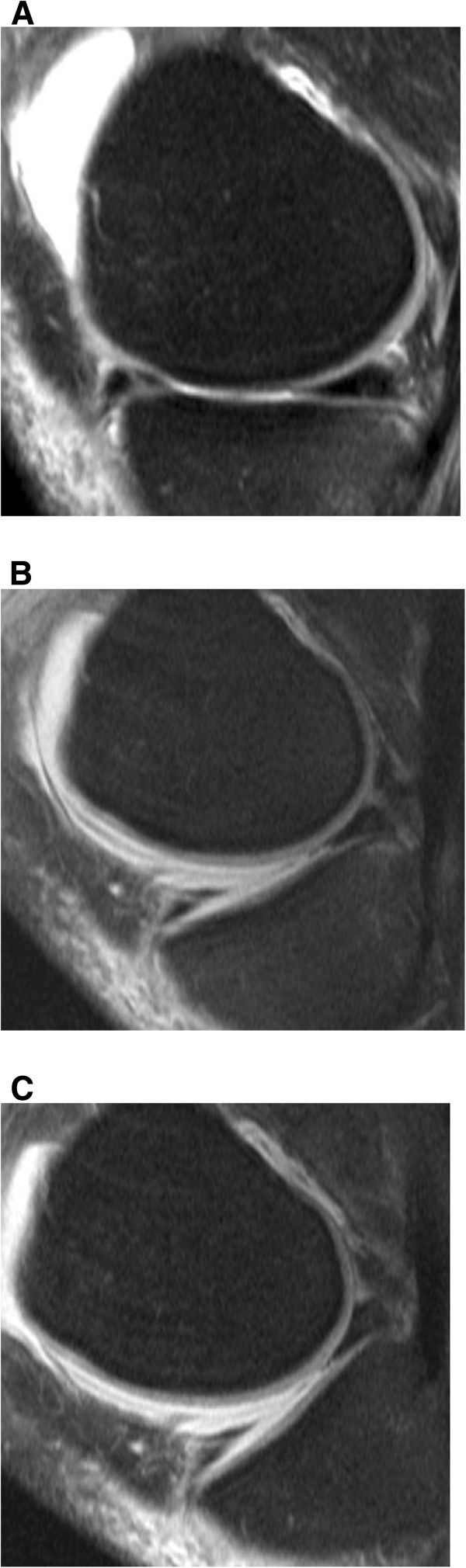
**Oblique vertical tear of the anterior horn of the medial meniscus.** T2-weighted TSE FS sagittal image (2900/90) obtained at knee extension (**A**), 30° of knee flexion (**B**) and 55° of knee flexion (**C**) demonstrate the oblique vertical tear extending from the surface to the undersurface of the medial menisci. No differences are recognized in the delineation of the tear at all three knee positions.

Arthroscopically, a partial torn ACL was present in eight of 29 patients (27.5%), and a complete ACL rupture was seen in 20 patients (69%), (Figure 3, Figure 4, (Table 3). In one patient, who had undergone arthroscopy (3.5%), an intact ACL was verified. At arthroscopy, a medial meniscus tear was diagnosed in 11 of 29 patients (38%), (Figure 5, Table 4). In 5 patients (17%), degeneration of the medial meniscus was verified, and in 13 of 29 cases (45%), the medial meniscus was diagnosed as normal. At arthroscopy, a lateral meniscus tear was diagnosed in 11 of 29 patients (38%). In two patients (7%), degeneration of the lateral meniscus was present. In 16 patients (55%), the lateral meniscus was evaluated as being intact. Tables 3 and 4 present the results of the comparison of MRI at versus arthroscopy in the diagnosis of ACL and meniscus injuries.

**Table 3:** **MRI Sensitivitiy compared to arthroscopy in the diagnosis of ACL injuries**

	Partial torn ACL	Complete ACL rupture
Arthroscopy	8/29	20/29
MRI 0° vs. arthroscopy	4/8 (50%)	17/20 (83%)
MRI 30° vs. arthroscopy	5/8 (63%)	17/20 (83%)
MRI 55° vs. arthroscopy	5/8 (63%)	19/20 (93%)

Diagnosis of these injuries by MRI was compared by arthroscopy. The number of correctly diagnosed ACL injuries and the percentage of sensitivity are presented. In one patient an ACL tear was diagnosed on MRI but couldnt be confirmed at arthroscopy. Because in only patients with suspected ACL tears seen on MR imaging, arthroscopies were performed, in this table the specificity of arthroscopically proven intact ACL can not be given.

Abbreviations:

ACL = Anterior Cruciate Ligament.

Vs. = versus.

**Table 4:** **MRI Sensitivitiy compared to arthroscopy in the diagnosis of meniscal injuries**

	Intact medial meniscus (specificity)	Medial meniscus tear (sensitivity)
Arthroscopy (n = 29)	13/29	11/29
MRI 0° vs. Arthroscopy	12/13 (92%)	8/11 (73%)
MRI 30° vs. Arthroscopy	12/13 (92%)	7/11 (64%)
MRI 55° vs. Arthroscopy	12/13 (92%)	8/11 (73%)

Diagnosis of these injuries by MRI was compared to arthroscopy. The number of correctly diagnosed healthy menisci (specificity) and pathological meniscal injuries (sensitivity) are presented.

## Discussion

Few studies have evaluated the effect of knee flexion in the visualization of the normal ACL and in suspected ACL tears on MR images. In a study by Niitsu and coworkers, diagnosis of ACL injuries was performed on 43 patients following MR imaging at knee extension and at 45° flexion (Niitsu et al. 1996). In this study the femoral ACL end was detected better in 53% of patients in an extended knee position compared to a flexed knee position. MR images with knee flexion provided a superior diagnosis in 48% of patients with disrupted ACL fibers and in 52% of residual torn ACL bundles. In Niitsu’s study, the patients were placed in a supine position surrounded by a mobile knee brace inside a flexible surface coil with knees in an extended or 45° position. A sophisticated positioning device such as ours was not utilized in this previous study. The same evaluation criterias was applied in Niitsu and our study. However, in Niitsu’s study the MR images were evaluated retrospectivly following arthroscopy and of the surgical results were known. An analysis of the MR images without knowing the arthroscopy findings was not carried out.

In an additional study, Pereira et al. examined 17 healthy subjects and 5 patients with a suspected ACL injury (Pereira et al. 1998). The examination was carried out at knee extension and with a slight knee flexion of approx. 17°. The study took place inside a 1.5 Tesla MR scanner using a small knee cushion to provide the flexion. Although the knee was only minimally flexed, the femoral ACL portion was better discerned in nearly every patient in response to knee flexion. In this small patient group, knee flexion was superior for detection of the torn cruciate ligament portions, and other knee structures, in three of five of patients (60%). Pereira et al. reported a sensitivity of 96% using MR in the flexed knee position when compared to arthroscopy.

However, no comparison between an extended and flexed knee position was performed. Therefore the diagnosis of intact or torn anterior cruciate ligaments was made only from the summary of both MR imaging studies.

In our study we found that MR evaluation of the central ACL portion was equally easy at knee extension and in response to 30° or 55° of knee flexion. A better evaluation of the tibial end of the ACL was observed at 55° of knee flexion compared to extension in 65% of patients (Niitsu et al. 1996; Pereira et al. 1998).

The reasons for better visualization of the ACL on MR images at knee flexion can be explained by several reasons. In vivo, as our studies showed, the femoral end of the ACL encompasses an increasingly horizontal orientation with increased flexion. This phenomenon is created by a tensioning of the anteromedial bundles in response to a simultaneous relaxation of the posterolateral bundles. In contrast, a relaxation of the anteromedial bundle and tightening of the posterolateral bundle occurs with further knee extension. With an increased flexion of the knee, the femoral cruciate ligament portion moves away from the intercondyle roof, so that ruptures in this area can be detected better, particularly on sagittal MRI scans. In addition, the femoral ACL end changes its shape at knee flexion. A flat-fanned shape in response to extension turns into a cylindrical shape with an increased flexion. In addition, with increased knee flexion, these structural changes of the femoral end of the ACL have the effect that the torn ligament structures can be better seen.

In an additional study by Lee et al. static MR images and arthroscopy were compared in the diagnosis of ACL ruptures at knee extension. This study reached a sensitivity of 94% and a specificity of 100% (Lee et al. 1998). However, no MRI examinations were carried out at flexion. In two other studies by Umans et al. and Yao et al., examinations were carried out in the evaluation of partial ACL ruptures (Umans et al. 1995; Yao et al. 1995). In these studies, arthroscopy also served as the gold standard. In the diagnosis of partial ACL ruptures, Umanns et al. reached sensitivities between 40% and 75% and specificity rates between 62% and 89%. In the study by Yao et al. 19-62% of the partial ACL tears and 90-94% of the complete ACL tears were detected.

In our study, the sensitivity in response to knee extension for partial ACL ruptures was 50% and 83% for complete ACL ruptures. In response to 30° of flexion and to 55° of flexion, the sensitivity of a partial rupture increased to 63% at both angles. Sensitivity of diagnosis of complete ruptures at 30° was 83 %. At 55° of knee flexion, the detection rate for complete ACL ruptures increased to 93%. However, we were not able to improve the sensitivity for partial ACL ruptures to 98%, which Heuck et al. published in his study using sagittal turbo-spin echo sequences (Heuck et al. 1994).

MRI examinations to compare the detection of meniscal tears at extension and different flexion positions were first published by Niitsu and coworkers (Niitsu et al. 2000). In this study a tear of the medial meniscus was verified arthroscopically in 17 cases and a tear of the lateral meniscus was seen in 10 patients. A sensitivity of 82% for the detection of meniscus tears was reached when images were obtained at either extension or 45° flexion of the knee. The specificities were 93% in response to extension and 99% in response to 45° of flexion. It was not possible to observe significant differences between knee extension and bending.

In our study, meniscus lesions were verified arthroscopically in 11 cases. It was not possible to find significant differences in the detection of medial or lateral meniscus tears when comparing knee extension to 30° or to 55° of knee flexion. The sensitivity for detecting medial meniscus tears was 73% at knee extension, 64% at 30° of flexion, and 73% at 55° of flexion. The specificities were 89% at extension, 92% at 30° of flexion and 89% at 55° of knee flexion. Lower sensitivities resulted for lateral meniscus tears. In all three knee positions, the sensitivity was only 55%. This low sensitivity for lateral meniscus tears can be compared to the study by Niitsu et al., who reported of a sensitivity of 48% for lateral meniscus tears in response to flexed and extended knees (Niitsu et al. 1991). The specificities with reference to an intact lateral meniscus were 97% for all three knee positions at extension, 30° and 55° of knee flexion.

In a study of more than 400 patients using a standard MR knee coil, De Smet et al. were able to verify a sensitivity of 93% for medial meniscus tears and of 80% for lateral meniscal tears (De Smet & Graf 1994). Justice and Quinn reported of sensitivities of 96% for the diagnosis of medial meniscal injuries and of 82% for lateral meniscal tears (Justice & Quinn 1995). It was noticeable that in particular the MRI diagnosis of lateral meniscal tears showed a high number of false negative findings. In the presence of a meniscal tear, a diagnostic accuracy of only 55% was reached independent of the knee position.

The reasons for this are manifold (De Smet & Graf 1994; De Smet et al. 1994; Kreitner et al. 1998). For instance, small peripherally located meniscal tears can lead to a misinterpretation between a fraying on the surface of the meniscus edge and a tear formation. A further reason for the low sensitivity of meniscal tears as detected in our studies can be seen in that sagittal as well as coronary sequences T1- and T2-weighted sequences combined with proton-density weighted images are normally used in standard MRI examinations. This combination of different imaging planes and a combination of sequences leads to an increased diagnostic accuracy in the evaluation of meniscus tears. A sagittal T2-weighted turbo spin echo sequence, however, was only acquired in our study for the diagnosis of meniscal tears.

In conclusion, MR Imaging of the knee at 55° of flexion and less at 30° of flexion allow an improved diagnosis of injuries to the anterior cruciate ligament as compared to MRI examinations at extension. The diagnosis of meniscal injuries, however, was not superior at either flexion positions compared to commonly performed examinations at knee extension.
